# Discovery and optimization of a synthetic small protein domain targeting antibodies

**DOI:** 10.3389/fbioe.2025.1678111

**Published:** 2025-11-12

**Authors:** Ana Margarida Gonçalves Carvalho Dias, Manuel João Brandão Matos, Cátia Soares, Carolina Natal, Ana Sofia Pina, Ana Cecília Afonso Roque

**Affiliations:** 1 Associate Laboratory i4HB - Institute for Health and Bioeconomy, Chemistry Department, School of Science and Technology, NOVA University of Lisbon, Campus Caparica, Caparica, Portugal; 2 UCIBIO – Applied Molecular Biosciences Unit, Department of Chemistry, School of Science and Technology, NOVA University Lisbon, Caparica, Portugal

**Keywords:** phage-display, rational design, protein engineering, tandem domains, WW domains

## Abstract

**Introduction:**

Antibodies and their derivatives constitute a crucial class of molecules in modern biotechnology and therapeutic development. Consequently, identifying chemically robust affinity ligands capable of specifically recognizing antibodies remains an important challenge.

**Methods:**

In this study, an in-house phage display library based on the WW domain scaffold (WWp5_4) was utilized to identify binders against polyclonal human IgG. A lead ligand from clone E6 was selected. To enhance the developability of WW ligands derived from this library, we rationally minimized structural liabilities within the scaffold framework to improve chemical stability and solubility, generating two mutant variants. Furthermore, a head-to-tail dimeric version of the E6 mutant sequence was designed.

**Results:**

The lead ligand E6 exhibited a dissociation constant of 133 nM. The mutant variants demonstrated a fivefold increase in expression yield compared to the native sequence. Additionally, the dimeric construct showed improved solubility and an estimated dissociation constant of 62 nM toward human IgG.

**Conclusions:**

This study underscores the potential of small folded protein domains, such as WW domains, as versatile affinity ligands for antibodies and other molecular targets, thereby broadening their applicability in biotechnology and bioengineering.

## 1 Introduction

Protein engineering is a potent instrument in the identification and conceptualisation of peptides and proteins that exhibit unprecedented designs (Jiang et al., 2024) and enhanced function, namely, binding against novel targets, and enhanced thermal and chemical stability (Dias and Roque, 2017; Arnold, 2018; Jumper et al., 2021). A widely adopted strategy in protein engineering is directed evolution, which employs *in vitro* techniques—such as phage display—to identify new affinity binders from existent libraries displaying peptides and proteins (Matos et al., 2020; Kruljec and Bratkovič, 2017). For instance, Nascimento *et al* employed a 12-mer phage display library to screen for peptides that specifically bind to the kappa light chains of Fab fragments (Nascimento et al., 2019). Similarly, Khan *et al* employed a T7 phage display library to identify peptides with affinity for avian IgY (Khan et al., 2017). Both examples share the common feature of short ligand length, which often results in peptide ligands with limited structural stability and poor folding. Moreover, to biological production of small peptide ligands is challenging, frequently requiring synthetic approaches such as solid-phase peptide synthesis. Besides, for therapeutic applications, small peptide ligands can be less chemically stable, prone to enzymatic degradation and low pharmacokinetic properties. In contrast, protein-based ligands—such as affibodies, affitins, DARPins, and repebodies—offer enhanced chemical stability, are amenable to biological expression and have improved pharmacokinetic properties thereby overcoming peptide-based ligands limitations. The prime example of a protein-based ligand that recognizes antibodies is the Protein A from *Staphylococcus aureus* (SpA), which has been extensively engineered in various formats. A Protein A domain led to the synthetic scaffold Z domain with about 58 amino acids, which was further engineered to improve the chemical stability of the protein against harsh pH conditions or the production of tandem sequences to increase the avidity against the target (Kanje et al., 2020).

Here, we exploit the potential of a synthetic small protein-scaffold derived from the WW domains, which are naturally occurring domains responsible for molecular recognition in diverse cellular pathways - WWp5_4 (Dias et al., 2025) - to discover new affinity ligands to bind full-length human antibodies. The WWp5_4 scaffold, with a size of 4.4 kDa, is positioned at the frontier between peptide and protein size, thereby combining advantages of both protein and peptide ligands. Thus, it can be considered as a robust mini protein (Lindner et al., 2024; Pham and Thomas, 2024). By combining protein engineering strategies–directed evolution for binding discovery and design for framework optimization - we developed the smallest antibody binding protein with affinity in the nM range. This workflow can be adapted and applied to other new binders selected from the new WW domain library or from small protein scaffolds.

## 2 Materials and methods

All used reagents were of the highest quality available and molecular biology grade. The full detailed list of materials and reagents used can be found in Supplementary Material.

### 2.1 Phage display biopanning

The description of the methods related to the amplification of helper phage, phage precipitation and pre-panning can be found in Supplementary Material. The first step of the phage selection began with the immobilization of target molecule, human polyclonal IgG, in the wells of the high binding 96-well microplate Corning 3,690 (Corning). 100 µg/well of the target was dissolved in 0.1 M sodium bicarbonate pH 8.6 and coated to the well of the microplate overnight at 4 °C. The biopanning conditions were varied during 6 panning cycles to increase the stringency, four different target concentrations and increasing number of washes were performed, which are summarized in Supplementary Table S1.

After coating, the 96-well plate was incubated with 150 µL of 3% (w/v) BSA in TBS (blocking solution) during 1 h at 37 °C. Then, 50 µL of the sterile phage was incubated during 2 h at RT. After removing the solution, 150 µL of TBS-Tween (washing solution) was added into each well and left incubating for 3 min. The number of washes was different in each round according to Supplementary Table S1. The washing solution was removed and the wells were rinsed with Ultra High Pure Water to remove residual TBS-T. Then, 50 µL of 100 mM Glycine at pH 2.2 (elution buffer) was added and incubated during 10 min at RT. The eluted phage was then immediately neutralized with 2M of Tris at pH 9.6 and used to infect an exponential 2 mL culture of *E. coli* ER2738 during 15 min at 37 °C, 250 rpm. At this point, a 10 µL sample was taken for the output titration. After that, 6 mL of LB with ampicillin (final concentration 20 μg/mL) was added to the culture and transferred to a 50 mL Falcon tube. The culture was incubated during 45 min, 37 °C, 250 rpm and after that, ampicillin to reach a final concentration of 50 μg/mL was added. Culture was incubated for an additional hour at 37 °C, 250 rpm. After that, 91 mL of LB with ampicillin (50 μg/mL concentration) was added to the previous culture as well as 1 mL of the helper phage VCSM13 stock solution (Invitrogen). The solution was transferred to a 500 mL Erlenmeyer flask and cultured for 1.5 h at 37 °C, 250 rpm. After that, kanamycin was added to reach a concentration of 70 μg/mL and left incubating overnight at 37 °C, 250 rpm. The protocol was repeated for all 6 rounds of panning. For the final round a non-amber suppressor strain was used, *E. coli* TOP10F′, instead of *E. coli* ER2738.

To understand the evolution of the panning, and if the ligands are being enriched, the determination of the titers of the input (phages amplified from the previous round of output) and output phages (eluted phages after panning) was performed. For that purpose, the sample from the input titration is serial diluted (10^4^ to the 10^13^) in LB and incubated with 50 µL of exponentially grown *E. coli* ER2738 cells, for 15 min at RT. After that, the culture was plated onto LB ampicillin plates and incubated overnight at 37 °C. The output titration was also serial diluted (10^2^ to the 10^6^) in LB and plated immediately onto LB ampicillin plates. The plates were incubated overnight at 37 °C and counted the next day for the number of isolated colonies. This procedure is repeated for all rounds of phage display panning (Supplementary Table S2). The output of the sixth round of panning was screened against the target to identify the lead ligand E6, details are provided in the Supplementary Material - methods section.

### 2.2 Expression and purification of lead sequence E6

The gene corresponding to the chosen E6 sequence was cloned according to the detail information in Supplementary Material. The bacterial clones with the insert DNA were selected for protein expression in *E. coli* Rosetta (DE3) cells. For this purpose, the DNA was purified with NZYMiniprep kit and was used for transformation onto *E. coli* Rosetta competent cells following the protocol of the manufacturer. For the protein expression, a pre-inoculum was prepared adding 5 µL of the transformed colonies glycerol stock in 5 mL LB medium containing ampicillin (vector resistance) and tetracycline (strain resistance). After 6 h of incubation, 1 mL of the culture was added to 100 mL of LB medium and was incubated overnight at 37 °C at 225 rpm. On the following day, 10 mL of the culture was inoculated onto 1 L of LB medium and was incubated at 37 °C at 225 rpm. When the optical density (OD_600nm_) reached 0.6–0.8 the culture was induced with IPTG (1 mM final concentration) and the temperature was lowered to 30 °C. After 20 h of protein expression, the culture was centrifuged (4,000 x g, 15 min, 4 °C) and the pellet was resuspended in 25 mL of appropriate buffer for further purification. As part of the biological production optimization supplementation tests on the medium culture were studied and are summarized in Supplementary Table S3. The resuspended pellet was subjected to 3 freeze/thaw cycles (−80 °C/RT) and then mechanical cell lysis was performed with 3 cycles of French Press (maximum 20,000 psi). The suspension was incubated with 10% (w/v) DNAse I on ice for 30 min and then centrifuged (10,000 x g, 30 min, 4 °C).

The supernatant corresponding to the soluble fraction was further purified by ion exchange and size exclusion chromatography, as detailed in Supplementary Material. The analytical methods for protein quantification and gel electrophoresis are also detailed in Supplementary Material.

### 2.3 Recombinant expression of mutant and dimeric sequences

The sequences WWp5_4 library and clone E6 sequences were engineered in the framework to reduce liabilities and generate two novel sequences - WWp5_4_M2 and E6_M2. The E6_M2 sequence was also used to generate a tandem sequence - the head-to-tail dimer E6_M2_T2. The comparison between the sequences and their estimated bio-chemical properties were calculated with Expasy Protparam (Supplementary Table S4) and analysis of physicochemical liabilities (Supplementary Table S5).

The WW domains genes were cloned into pET21a (Histidine tag in C-terminal position) between NheI and XhoI and transformed in *E. coli* Rosetta (DE3). The expression protocol was the same as described for lead sequence E6, but with some modifications. Briefly, the protein expression occurred in 100 mL to test in ELISA or 1 L for purification and biophysical characterization. In both strategies the media culture used was Terrific Broth media (24 g/L Yeast Extract, 20 g/L Tryptone, 4 mL/L Glycerol, 100 mL/L Phosphate buffer (0.17 M KH_2_PO_4_, 0.72 M K_2_HPO_4_ pH 7.4)) with ampicillin (100 μg/mL, stock 100 mg/mL). This culture was incubated at 37 °C, 220 rpm until OD_600nm_–0.8, after that, it was induced with 1 mM IPTG and supplemented with 200 mM Arginine (stock 2M Arginine at pH7.5, pH was adjusted with acetic acid) and continued the expression at 30 °C, 220 rpm for overnight. In the next day the culture was harvested by centrifugation 10 min, 4,000×g at 10 °C, the pellet was collected and lysed using 1 g of pellet/5 mL of Lysis buffer (PBS 1x, 0.2 mg/mL Chicken Lysozyme (Sigma-Aldrich) and DNAseI (Roche)) with vigorous shaking for 1 h at room temperature. The extract was centrifuged 8,000 *g* for 20 min at 4 °C and the soluble fraction was collected. The fractions were quantified by BCA assay and analysed by gel electrophoresis as described previously.

### 2.4 Chromatographic purification of E6 mutant and its dimeric form

Since E6M2 and E6_M2_T2 had an His-tag they were purified by immobilized metal affinity chromatography (IMAC). Therefore, after expression the soluble fractions were further centrifuged at 42,000 *g* for 20 min at 4 °C. As the samples were diluted (∼5 mg/mL in 25 mL lysis buffer), the soluble fractions were lyophilized and resuspended in 4 mL of water. The samples were dialyzed (Membranes Spectra Pre-Treated 1 kDa, Carl Roth) to Binding buffer without imidazole (20 mM Sodium Phosphate, 500 mM Sodium Chloride pH 7.4) for 24 h at 16 °C. The samples were quantified by BSA assay (typically ∼6 mg/mL), this solution was diluted 1:2 in Binding buffer (20 mM Sodium Phosphate, 500 mM Sodium Chloride, 20 mM imidazole pH 7.4) and purified in a gravitational flow with 6 mL of resin (IMAC Sepharose 6 Fast Flow (Ni^2+^), Cytiva) packed in a PD-10 column. The column was prepared using the indications of the manufacturer. The binding of the protein to the column was maximized by an incubation for 1 h at room temperature in agitation at 40rpm. The resin was washed 4 x column volumes (CV) with binding buffer. The elution as achieved by incubation for 15min with elution buffers. We used a gradient with 3 x CV elution buffer 1 (binding buffer with 100 mM imidazole) and 3 x CV elution buffer 2 (binding buffer with 200 mM imidazole).

The samples were analysed by BCA assay for that the samples were precipitated in ethanol and dissolved in 5% (v/v) SDS in 0.1 N NaOH. As well the protein samples were analysed in a tris-tricine gel with 20 µg total protein/lane. The gels were stained with silver staining and documented in GelDoc.

The most enriched elutions in WW domains (60%–70% purity) were combined and dialysed against PBS1x (10 mM Na2HPO4, 1.8 mM KH2PO4, 2.7 mM KCl, 137 mM NaCl pH 7.4) with routine changes of buffer up to 48 h at 16 °C. Following the samples were concentrated with a Amicon-Ultra15 3 kDa MWCO (Millipore) with centrifugation conditions 4,900 g, 30 min, 4 °C. The final samples were quantified by BCA assay.

Size Exclusion Chromatography (SEC). The samples were further purified by SEC to achieve 90% purity in a AKTA system (Cytiva). The column Superdex 75 Increase 10/300 GL (29148721, Cytiva) was washed with water and equilibrated 2CV in PBS1x, as described by the manufacturer. As calibrants we prepare a mixture: 2 mg/mL of Bovine Serum Albumin (66 kDa, BP9702-100, FisherScientific), Ovalbumin (42 kDa, A5503-1G, Sigma), Chicken Lysozyme (18 kDa, 62,971–10G-F, Fluka) and hPin1_WW chemically synthesized (5 kDa, produced in (Dias et al., 2015)) in PBS 1x, this sample was filtered with 0.2 µm PES (514–0073, VWR) and analysed. The operation conditions: flow 0.5 mL/min, 0.05 CV in PBS1x and injection of 250 µL and run for 2 CV with PBS 1x at 16 °C. The same method was used for the WW domain samples. After purifications samples were quantified for total protein by BCA assay, as described previously and used for characterization.

### 2.5 Characterization of E6 binders

#### 2.5.1 Enzyme-linked immunosorbent assay (ELISA)

The soluble fraction of 100 mL cultures (100 μL) was used for ELISA against hIgG, as described in (Dias et al., 2025). Briefly, the target hIgG was immobilized (1 μg/well) in 0.1 M Sodium Bicarbonate pH 8.6 in Immuno Maxisorp ELISA plates (Thermo Fisher), overnight at 4 °C. In the next day, the unbound hIgG was removed, and the wells washed with PBS1x. To the wells was added the blocking solution – 5% (v/v) Soya Milk in PBS 1x and incubated for 1 h at room temperature. The wells were washed and incubated with 100 µL of soluble fraction (∼0.065 mg/mL total protein) at room temperature for 2 h.

The unbound WW domains were removed, and the wells were washed using 4 times PBS1x-0.05% Tween 20 and 1 time with PBS1x. For detection was used 1:1,000 anti-His-HRP conjugated antibody (MA1-21315-HRP, Invitrogen) in 1% (v/v) Soya Milk in PBS1x and incubated for 1 h at RT, after that the wells were washed with 5x PBS1x. The detection occurred with ABTS (2,2′-azino-bis(3-ethylbenzothiazoline-6-sulfonic acid) ready solution (11684302001, Roche), which was incubated in the dark for 1 h at 37 °C. Finally, the results were analysed in a microplate reader (Tecan) with appropriate filter at Abs_405nm_.

#### 2.5.2 Microscale Thermophoresis (MST)

For MST assays of E6, IgG was conjugated with fluoresceine isothiocyanate (FITC) using standard protocols. The conjugated IgG-FITC was used at a fixed concentration of 50nM and 16 serial dilutions of E6 protein (from 23.5 µM to 0.7 nM) were made in 50 mM MOPS 0.05% (v/v) Tween-20 pH 7.

For MST assays of E6_M2_T2, the target was hIgG-FITC (F9636, Sigma) at a constant concentration of 25 nM. For the E6_M2_T2 ligand, 16 samples were prepared in variable concentrations starting from 695 nM and using a serial dilution of 1:2 up to a minimum concentration of 25 nM using PBS1x-0.05% (v/v) Tween-20 pH7.4.

Samples were transferred to a standard capillary (Monolith NT.115 Capillary, Nanotemper) with the following settings: medium MST-power, excitation power at 20%, with excitation in Nano-Blue, with thermostat at 25 °C. The signal of the samples was read in triplicates using a Monolith NT.115 (Nanotemper) and the signal was processed in the manufacturer software with FitModel: Kd and Hill. The binding parameters (K_D_ and EC_50_) and were estimated automatically in the MST software.

#### 2.5.3 Attenuated total reflectance–fourier transform infrared spectroscopy (ATR-FTIR)

After size exclusion purification, solutions of 5 μg/mL E6_M2 and 6 μg/mL E6_M2_T2 in PBS1x were analysed by ATR-FTIR. The spectrum was recorded using Spectrum Two with UTAR two adapter from Perkin Elmer. Scans were recorded at room temperature using the following parameters: absorption mode; with background air; 25 scans were obtained and averaged in the range between 4,000 and 400 cm^−1^.

#### 2.5.4 Molecular modelling of WW domain structures

WW domain sequences presented in this work were modeled using AlphaFold3 (Jumper et al., 2021; Varadi et al., 2024), the fasta sequence of WWp5_4 with loops substituted with alanines in the loops (Loop I - 5 alanines and in Loop II – 4 alanines), E6, WWp_5_4_M2, E6_M2 and E6_M2_T2 were provided as input, and the best ranked model had a pLDDT over 77, which is associated with a good model for the backbone.

## 3 Results and discussion

### 3.1 Discovery and selection of a new biological ligand for human IgG

**FIGURE 1:**
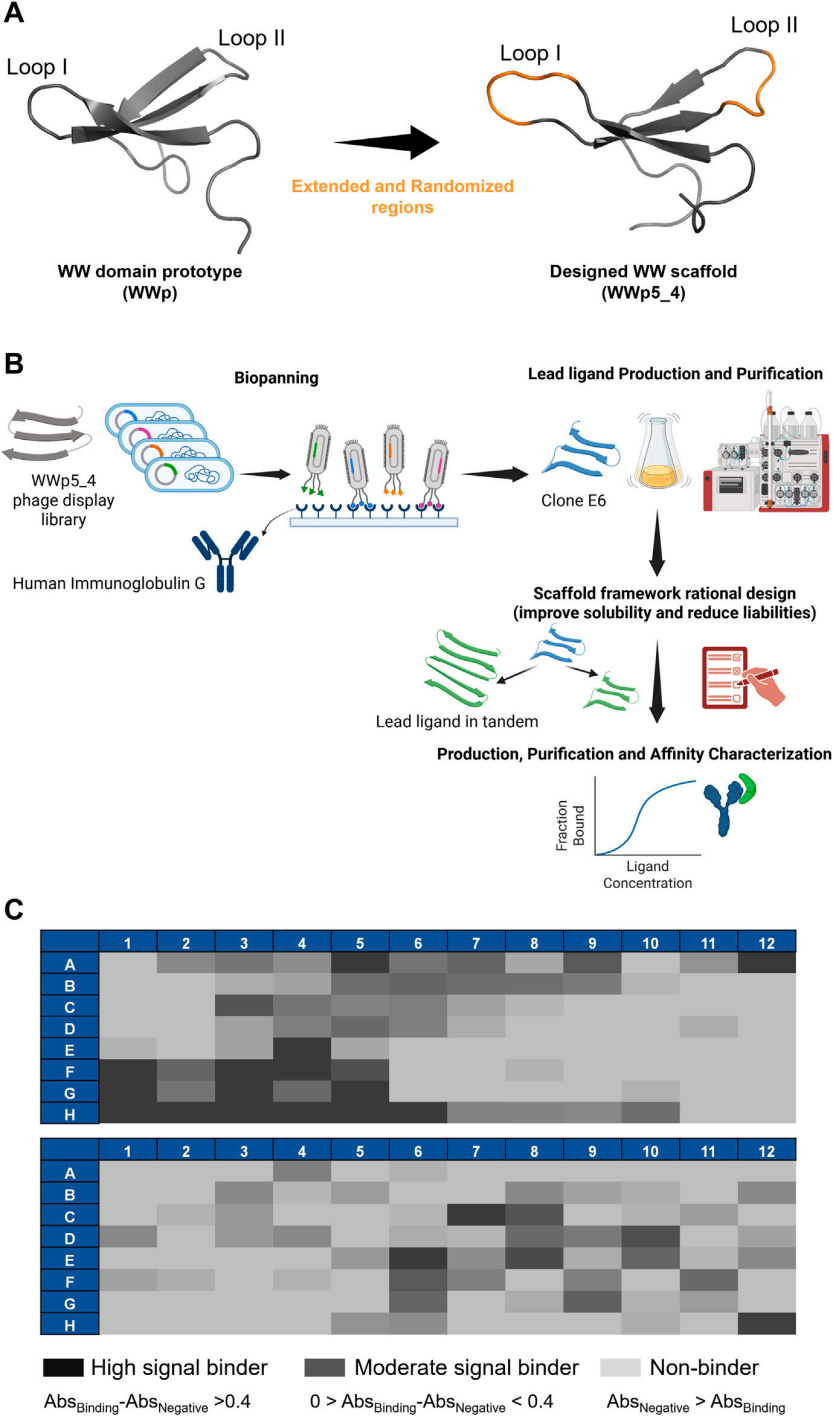
Research strategy and biopanning results. **(A)** Representation of the WW domain prototype sequence (WWp, PDB:1E0M (Macias et al., 2000)) and subsequent WWp5_4 domain scaffold used in the phage display library. The WWp5_4 model structure was generated in AlphaFold3 with an average pLDDT of 85.53, where in orange is identified the randomized loops and in grey is represented the framework maintained constant. Images were generated in Pymol software. **(B)** Research strategy used in this work. Dias, M. (2025) https://BioRender.com/a55s887. **(C)** ELISA results concerning the 192 clones screened through phage display for human IgG. The results for each clone are color-coded based on the difference in absorbance against the target versus the absorbance against the negative control (Human Serum Albumin).

**FIGURE 2:**
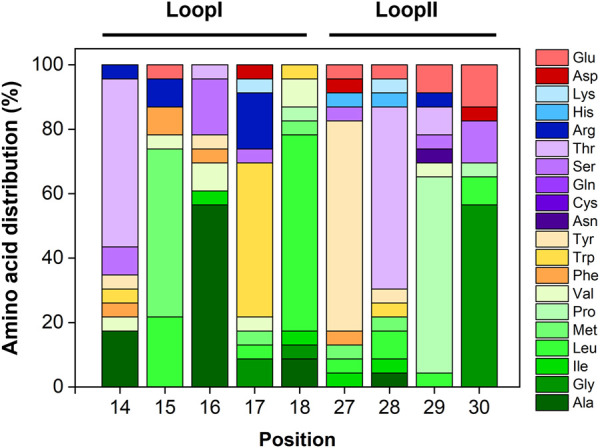
Sequence analysis of the most promising clones selected against human IgG. Relative amino acid distribution (in %) in Loops I and Loop II per residue. Loop I is located at positions 14–18 and loop II is located between residues 27–30. Repeated sequences were disregarded from this analysis.

The biopanning of the previously developed WWp5_4 library (Dias et al., 2025) (Figures 1A,B) against human polyclonal IgG was performed for six rounds, after which an enrichment of ligands was observed (Supplementary Table S3). To assess the binding of each clone to the target, 192 clones were selected and expressed at small scale in 96-deep well blocks, following conditions previously optimised. After cell lysis, the supernatant containing the soluble fraction was used for ELISA assays. The results in Figure 1C illustrate the difference in the absorbance profile of the screened ligands. The most interesting ligands should ideally exhibit high binding signal towards the target, while exhibiting lower values for human serum albumin, which was used as a negative control since it is a major contaminant in human plasma. In total, 63 clones presented higher binding signal towards IgG and from those, 17 were considered as high binders (Abs>0.4), which shows the successful panning strategy. Considering these results, the 26 most interesting clones (17 high signal and 9 moderate signal) were sent for Sanger sequencing. From these 26, only 21 presented high quality for reliable sequencing results. The sequence alignment and diversity of the different binders is represented in Table 1 and Figure 2A.

**TABLE 1:** Clones sequence diversity was compared with the WWp5_4 library framework derived from the WW prototype (PDB: 1E0M (Macias et al., 2000)) using Clustal Omega and Jalview softwares. X–represents any amino acid.

Clone	Sequence
WW näive library (WWp5_4)	SMGLPPGWDEYKT XXXXX GKTYYYNH XXXX KTSTWTDPRMSS
A5, A12, F5, H1, H2, H12, G1, F1, F3, H4	SMGLPPGWDEYKT TMAWL GKTYYYNH YTPG KTSTWTDPRMSS
H7	SMGLPPGWDEYKT ALSRW GKTYYYNH DWNP KTSTWTDPRMSS
H8	SMGLLPGWDEYKT WVVDV GKTYYYNH FKVS KTSTWTDPRMSS
G3	SMGLPPGWDEYKT SLSRP GKTYYYNH EEPS KTSTWTDPRMSS
H3	SMGLPPGWDEYKT YRVMV GKTYYYNH LYRL KTSTWTDPRMSS
H6	SMGLPPGWDEYKT VLYSG GKTYYYNH IMSL KTSTWTDPRMSS
C8	SMGLPPGWDEYKT SMALL GKTYYYNH YTPG KTSTWTDPRMSS
D10	SMGLPPGWDEYKT FEIVM GKTYYYNH MHTD KTSTWTDPRMSS
E6	SMGLPPGWDEYKT RRFKL GKTYYYNH HILS KTSTWTDPRMSS
G9	SMGLPPGWDEYKT TLAGL GKTYYYNH YTPG KTSTWTDPRMSS
G5, F6	SMGLPPGWDEYKT AFSRA GKTYYYNH YLEE KTSTWTDPRMSS
	LoopI LoopII

The output sequences from the sixth round of panning exhibited considerable variability in composition. Notably, a specific sequence—loop I: TMAWL and loop II: YTPG—was identified in 10 different clones, indicating a potential preference for this particular amino acid combination in target binding. Overall, analysis of the amino acid frequencies in the selected clones revealed that both loops are enriched in hydrophobic (aliphatic and aromatic) and polar uncharged residues (Supplementary Figure S1).

Figure 2B depicts the amino acid distribution across each loop position. In loop I, the most frequently occurring residues are alanine, leucine, methionine, tryptophan, and threonine. Valine, serine, and arginine also appear at equal frequencies (6%). For loop II, tyrosine, proline, and threonine dominate, followed by glycine. Notably, glutamic acid—a negatively charged residue—accounts for approximately 7% of the composition in this loop.

Selection of the lead ligand for further studies was based on multiple criteria, including sequence prevalence, ELISA-derived binding signals, loop-specific amino acid composition, and inherent solubility indices (Table 2). The WWp5_4 scaffold exhibits pronounced hydrophobicity, primarily due to the presence of two tryptophan and four tyrosine residues—three of which are consecutively located within the second β-sheet—as well as three terminal proline residues. To mitigate the risk of selecting overly hydrophobic clones, which could hinder bacterial expression, we prioritized clones featuring hydrophilic and charged residues within the loops to ensure a balanced overall hydrophilicity.

**TABLE 2:** Summary of the obtained ligands against human IgG in terms of their loops sequence, sequence repetition, intensity of ELISA signal, solubility index and isoelectric point (pI). Solubility index was estimated in Pepcalc online tool and pI was estimated in Expasy–Protparam tool (https://web.expasy.org/protparam/).

Clone	Loop I	Loop II	Sequence repetition	ELISA signal	Solubility index	pI	Selected for expression
A5	TMAWL	YTPG	10	High	Poor	8.02	Yes
H7	ALSRW	DWNP	1	Moderate	Good	8.05	Yes
H8	WVVDV	FKVS	1	Moderate	Poor	8.02	Yes
G3	SLSRP	EEPS	1	High	Good	6.49	-
H3	YRVMV	LYRL	1	High	Poor	9.45	-
H6	VLYSG	IMSL	1	High	Poor	8.02	-
C8	SMALL	YPTG	1	Moderate	Poor	8.02	-
D10	FLIGM	MHPY	1	Moderate	Poor	5.98	-
E6	RRFKL	HILS	1	High	Good	9.77	Yes
G9	TLAGL	YTPG	1	Moderate	Poor	8.02	Yes
G5	AFSRA	YLEE	2	High	Good	6.49	Yes

Considering these criteria, clones A5, H7, H8, E6, G5 and G9 were selected to further evaluate their biological production in small scale (Tris-tricine gels in Supplementary Figure S2). A clear band correspondent to the expected protein size was observed for A5, H8, E6 and G5 after induction, but not for H7 and G9. The sequence derived from clone H7 has a good water solubility prediction, but a high degree of hydrophobic residues (2 additional tryptophans, alanine and leucine) and proline residues (known for disrupting proteins secondary structure (Morgan and Rubenstein, 2013)), while the estimated solubility of clone G9 sequence is low, which may justify the poor expression levels observed.

Concerning clone E6, we observe a more intense band after induction when compared to the other clones. The apparent easiness in the biological production, allied to a strong ELISA signal and the presence of arginine and phenylalanine in the loops (typical in other IgG binders reported in the literature (Kruljec and Bratkovič, 2017)) justified the selection of E6 as the lead ligand to carry on our studies.

### 3.2 Optimising the production of the selected lead ligand E6

**FIGURE 3:**
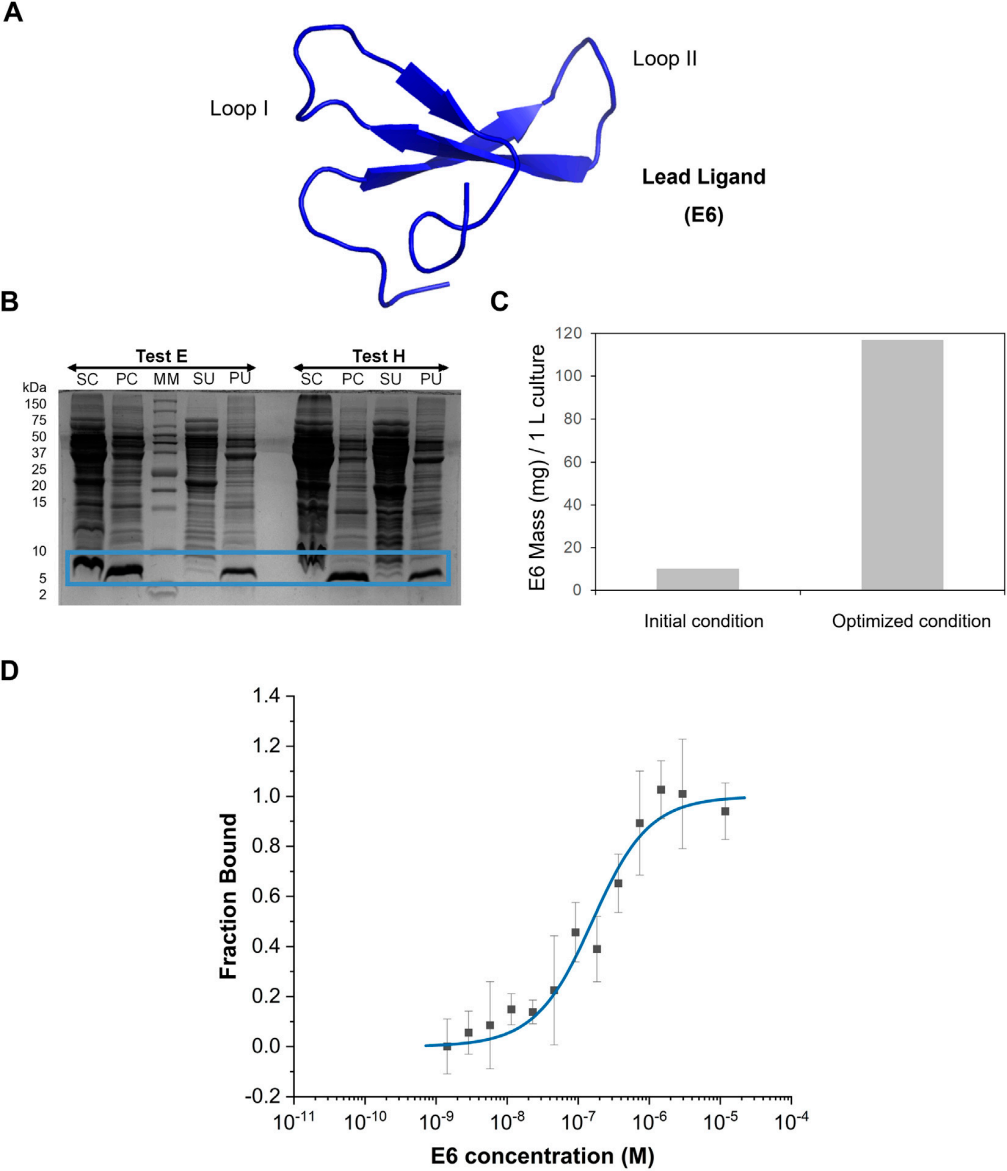
E6 protein production and characterization. **(A)** Representation of E6 structure generated in AlphaFold3 with an average pLDDT of 89.41. Image was generated in Pymol software. **(B)** Tris-Tricine gel regarding E6 partition between the soluble and the insoluble fraction in tests E (supplementation 200 mM L-arginine after induction) and H (supplementation 200 mM L-arginine and L-glutamic acid after induction). SC- Supernatant containing the soluble fraction after first centrifugation, PC-Pellet after first centrifugation, SU-Supernatant containing the soluble fraction obtained after ultracentrifugation, PU-Pellet after ultracentrifugation. MM–Protein Marker. **(C)** Comparison of the productivity obtained before and after optimization of E6 biological production (test E), based on the analysis of soluble fraction by total protein quantification and estimation of purity through SDS-PAGE. **(D)** Dose-response curve determined by Microscale Thermophoresis for the complex between E6 and IgG-FITC (n = 4). Y-axis represents the fraction bound of the human IgG-FITC.

The WWp5_4 scaffold is composed by 42 amino acid residues with a molecular weight of approximately 5 kDa. We have previously proposed successful methods for the chemical synthesis of WW domain peptides (Dias et al., 2015; Dias et al., 2016; Dias et al., 2025). It has also been shown that the biological expression of these proteins is possible when produced as fusion proteins, for example, with GFP (Pina et al., 2015). Here, we attempted the biological production of E6 ligand without any tag or fusion protein partner (Figure 3A) to minimize off target interactions. One of the strategies to improve the solubility of the recombinant protein expression in *E. coli* is the supplementation of amino acids into the culture medium (Maser et al., 2019; Kumar et al., 2020), namely, L-arginine, which has been described as a protein aggregation inhibitor and a promotor of protein refolding (Lange and Rudolph, 2009). The combination of L-arginine with L-glutamic acid has been shown to prevent WW domain proteins aggregation (Golovanov et al., 2004). Hence, the effect of amino acids supplementation during protein expression was studied (Supplementary Table S3). The protein expression profile was evaluated under normal expression conditions (without supplementation of amino acids, test A) and a strong band at ∼ 5 kDa is visible (Supplementary Figure S3A). Supplementation with increasing concentrations of arginine from 50 to 200 mM of amino acid (test C, D and E respectively) benefit the expression of E6 (Supplementary Figure S3B-D). The effect is more noticeably at 100 mM and 200 mM. Concerning the combined effect of L-arginine and L-glutamic acid (tests F, G and H), there is noticeable improved expression with higher amino acids concentration (Supplementary Figure S3B-D). To assess whether test E or test H has higher content of soluble E6, bacterial cells were lysed and the soluble supernatant analysed. The expressed protein is partitioned between the soluble (16%) and insoluble fraction, which shows the tendency for WWp5_4 scaffold proteins to be expressed as inclusion bodies (Figure 3B). Test E yielded the best results with a 12x increase in the productivity before and after optimization (Figure 3C). Finally, due to the absence of expression tags on protein E6 sequence, the purification scheme relied on ion-exchange chromatography (IEX). E6 was purified with a strong cation-exchanger resin at pH6 under denaturant conditions (4M Urea), which resulted in the successful enrichment of E6 with a final 47% purity (Supplementary Figure S4A-D). The protein was further purified by size exclusion chromatography (final purity >90%) (Supplementary Figure S5).

### 3.3 Binding affinity characterization of lead ligand E6

The affinity of E6 towards human IgG was measured using Microscale thermophoresis (MST) (Figure 3D). The EC_50_ was determined in 146 nM and the Hills coefficient was 0.95, suggesting a non-cooperative binding (Goutelle et al., 2008). The determined dissociation constant, K_D_, towards IgG was 133 ± 48 nM, which is within the range reported for other affinity ligands. Typically, peptides discovered by phage display have dissociation constants in the µM range, while engineered scaffolds (e.g., affibodies, affitins, darpins or repebodies) reach the low nM range, comparable to natural biological ligands such as SpA, SpG and PpL (Kanje et al., 2020).

### 3.4 Optimization of the scaffold sequence framework

Following work with the WWp5_4 naïve library, we found that lead ligand sequences—such as CW3S binding human serum albumin (Dias et al., 2025) and the E6 clone developed in this study—exhibited poor expression, challenging purification, and limited solubility. Consequently, it became necessary to optimize the WWp5_4 scaffold framework to enhance solubility and minimize liabilities, which is critical for various applications including bioseparation, *in vitro* diagnostics, and therapeutic development (Wu, 2019).

From our previous work with human Pin1 (hPin1_WW (Dias et al., 2015)) we observed that this sequence was more soluble in aqueous media and easily processed (Table 3). Also, we observed that the expression of hPin1_WW in fusion with GFP was quite successful with a yield of 1840 mg/L (Pina et al., 2015). These observations are further corroborated by physicochemical analysis with online tools (PepCalc https://pepcalc.com/and Marvin software; analysis summarized in Table 3). The sequence alignment of hPin1_WW and WWp5_4 highlights the higher hydrophobicity of the WWp5_4 framework and predicted higher Aliphatic Index and LogP for hPin1_WW versus WWp5_4. These complicates recombinant expression in bacterial hosts and further ligand developability (Chiu and Gilliland, 2016; Chiu et al., 2019).

**TABLE 3:** Protein modifications introduced in the WW prototype framework regions, and also on the selected IgG binders. Loop region is underlined. The amino acid residues mutated are highlighted in red, and the new residues introduced are highlighted in blue. The N-terminal Cysteine is highlighted in green (note: this amino acid was not considered in the analysis). n.a. – not applicable. Physicochemical parameters were estimated by Expasy–Protparam (https://web.expasy.org/protparam/); LogP was determined in Marvin software. Aliphatic Index is calculated based on side chains of hydrophobic amino acids (Ala, Val, Ile and Leu), a higher value suggests a more thermostable globular protein. Log P (logarithm of the partition coefficient (P)) is estimated based on the ability of a compound to be soluble in n-octanol versus water. A lower Log P value indicates a higher solubility in water.

Target	Name	AA sequence	AA#	MW (Da)	pI	Extinction coefficient (M^-1^cm^-1^ at 280 nm)	Aliphatic index	Log P
Cognate peptides	human Pin1 WW domain (PDB: 2M8I)	MADEEKLPPGWEKRMSRSSGRVYYFNHITNASQWERPSG	39	4,599.09	8.21	13,980	32.56	−32.29
n.a	WW_Prototype PDB 1E0M, framework of WWp5_4 scaffold	S MG LPPGW D E Y KTHNG K TYYYNHNTKTS T W TD PR M SS	37	4,352.74	8.08	16,960	10.54	−36.46
	WW_Prototype_M2 (WWp5_4_M2)	S GK LPPGW E E R KTHNG R TYYYNHNTKTS Q W EH PR G SS	37	4,365.66	9.30	15,470	10.54	−36.26
Human IgG	E6	S MG LPPGW D E Y KTRRFKLG K TYYYNHHILSKTS T W TD PR M SS	42	5037.70	9.77	16,960	37.14	−30.54
	E6_M2	C S GK LPPGW E E R KTRRFKLG R TYYYNHHILSKTS Q W EH PR G SS	42	5050.63	10.11	15,470	37.14	−32.06
	E6_M2_T2	C S GK LPPGW E E R KTRRFKLG R TYYYNHHILSKTS Q W EH PR G SSS GK LPPGW E E R KTRRFKLG R TYYYNHHILSKTS Q W EH PR G SS	84	10,083.25	10.23	30,940	37.14	−51.29

**FIGURE 4:**
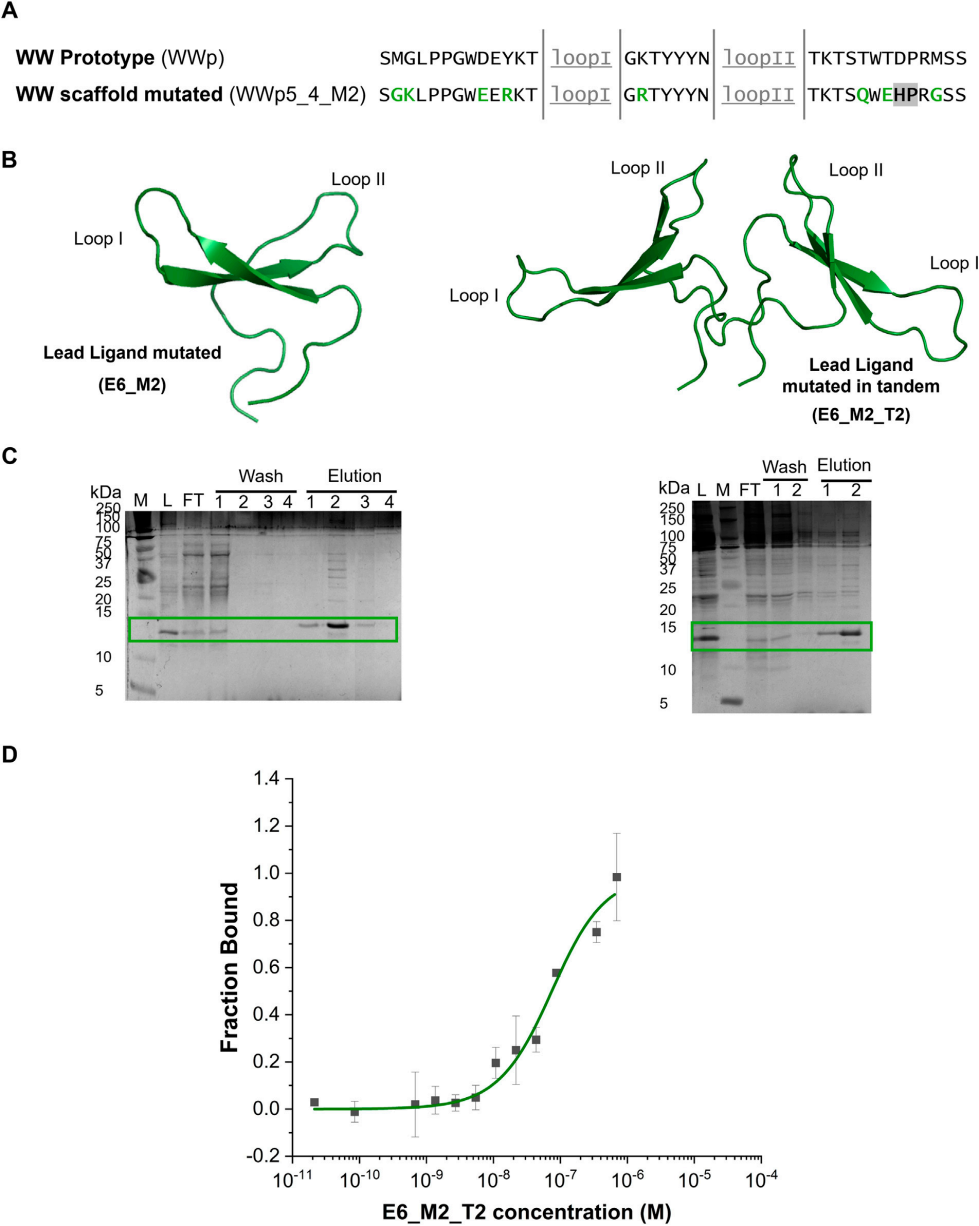
Sequence mutations strategy, production and characterization of the E6_M2 and E6_M2_T2 against human IgG. **(A)** Comparison between the WW prototype (PDB: 1E0M (Macias et al., 2000)) framework sequence and the mutations in the framework of WW domain scaffold. The loops are underlined (grey), the mutations to improve the solubility are highlighted in green and the mutation to eliminate chemical liability is highlighted in grey. **(B)** Representation of E6_M2 and E6_M2_T2 structures generated in AlphaFold3 with an average pLDDT of 89.64 and 77.91, respectively. The image was generated in Pymol software. **(C)** Tris-tricine gels of immobilized metal affinity chromatography purification of E6_M2 and E6_M2_T2, respectively. Both small proteins are separated in the gel with a band at ∼12.5 kDa. M - Precision Plus Protein™ Dual Xtra Prestained, Biorad, L–Loading; FT - Flowthrough. The mutants were eluted mainly in fraction 1 and 2. **(D)** Dose-response curve determined by Microscale Thermophoresis for the complex between E6_M2_T2 and IgG-FITC (n = 3). Y-axis represents the fraction bound of the human IgG-FITC.

To enhance the developability of ligands derived from the WWp5_4 scaffold, we minimized framework liabilities. This involved assessing the chemical and physical stability of the sequence, guided by established insights from antibody engineering. For chemical stability, we focused on regions susceptible to modification or degradation, including Asn deamidation, Met and Trp oxidation, Asp isomerization, and hydrolysis at AspPro/AsnPro motifs—processes known to occur within the pH range of 5.5–8 (Xu et al., 2019). Regarding the physical stability we looked for proteolysis potential sites, as well as very hydrophobic regions (Chiu et al., 2019). Tables 3, 4 summarize the sequence modifications performed on the WW prototype framework, as well as the physicochemical properties predicted for the newly designed sequences. Our rational design was validated by the theoretical analysis, as we reduced the number of chemical liabilities in the framework, while improving the solubility (higher LogP) for WWp5_4_M2 (Figure 4A). The same framework modifications were applied for the lead ligand E6 (as an example for lead optimization) originating the sequence E6_M2.

**TABLE 4:** – Analysis of physicochemical liabilities in the designed WW sequences. Loop region is underlined. The amino acid residues mutated are highlighted in red, and the new residues introduced are highlighted in blue. The N-terminal Cysteine is highlighted in green.

			Oxidation		Deamination (Asn, N)					Hydrolysis	
Name	AA sequence	AA#	M	W	NG	NNG	NT	NS	NY	NP	DP
WWp5_4	S MG LPPGW D E Y KTHNG K TYYYNHNTKTS T W TD PR M SS	37	2	2	1	0	1	0	0	0	1
WWp5_4_M2	S GK LPPGW E E R KTHNG R TYYYNHNTKTS Q W EH PR G SS	37	0	2	1	0	1	0	0	0	0
E6	S MG LPPGW D E Y KTRRFKLG K TYYYNHHILSKTS T W TD PR M SS	42	2	2	0	0	0	0	0	0	1
E6_M2	C S GK LPPGW E E R KTRRFKLG R TYYYNHHILSKTS Q W EH PR G SS	42	0	2	0	0	0	0	0	0	0
E2_M2_T2	C S GK LPPGW E E R KTRRFKLG R TYYYNHHILSKTS Q W EH PR G SSS GK LPPG W EE R KTRRFKLG R TYYYNHHILSKTS Q W EH PR G SS	84	0	4	0	0	0	0	0	0	0

In nature, WW domains are important molecular recognition domains and often appear in tandem sequences, which are indispensable for WW domain function and regulation (Huang et al., 2009; Lin et al., 2019; Rotem-Bamberger et al., 2022; Wenz et al., 2022). Besides, it is known that tandem structures increase the avidity and enable multivalent target recognition (Kruljec and Bratkovič, 2017; Chiu et al., 2019; Lin et al., 2019). This strategy was also applied to the Z domain, which is commercialized as a multimeric protein with 4–6 repeats (Kanje et al., 2020). Inspired by these strategies, we constructed a dimeric version of E6_M2 sequence–the E6_M2_T2. In the E6_M2 and E6_M2_T2 sequences we further introduced a Cys at the N-terminal, which leaves a sulfhydryl-reactive chemical group that can be used as a moiety to facilitate further modification: conjugation with a fluorophore or a protein; or immobilization in a solid-support, as previously described for hPin1_WW, which was immobilized in agarose beads using maleimide conjugation chemistry (Dias et al., 2015; Dias et al., 2016).

After establishing the new designs, the recombinant expression levels of the different sequences were compared (Supplementary Figure S6A). Firstly, the mutated framework design (WWp5_4_M2) produces about 4-times more protein than the initial framework (WWp5_4). Secondly, the selected IgG-ligand E6_M2 presents a band at approximately 12.5 kDa, which might be due to the presence of the N-terminal Cys residue that promotes the formation of stable dimers. We observed an approximate 5-times increase in the expression of soluble protein, when compared with the native sequence (E6) under the same expression conditions (Figure 3C).

Supplementary Figure S6B shows the binding signal against human IgG obtained by ELISA for the native and mutated sequences. E6_M2 presents a 2-times higher binding signal than the scaffold framework (WWp5_4), indicating that the mutations did not affect the molecular recognition capability of E6_M2. Besides, in Supplementary Figure S7 we compare the binding signal and expression signal between E6, E6_M2 and *E. coli* Rosetta extract (negative control, no protein expression). We observe a 1.5-times binding and expression signal for E6 when compared with the negative control, while for E6_M2 we observe a 2-times binding signal and 9.5-times the expression signal when compared to the negative control. Overall, these results indicate that the E6 sequence was successfully engineered, since we maintained the molecular recognition capability and increased the expression level. Encouraged by these results, we continued with the expression of the dimeric E6_M2_T2 and we observed a 5-times increase in protein yield for E6_M2. Furthermore, we observe a more reproducible expression level for E6_M2_T2 (about 623 ± 56 mg protein per 1L culture).

### 3.5 Characterization of optimized IgG ligand

The soluble fractions obtained for the proteins E6_M2 and E6_M2_T2 (Figure 4B) were purified by IMAC under standard conditions, since chaotropic agents were not required, indicating higher solubility than the native counterpart (lead ligand E6). Figure 4C shows the gel after IMAC purification and the estimated a purity of 60%. After performing dialysis, we observed some precipitation for E6_M2, while E6_M2_T2 was always soluble. Furthermore, E6_M2_T2 was more stable, and during the purification of this molecule by size exclusion chromatography we did not observe any precipitation, and determined a final purity of 90% (Supplementary Figure S8).

We proceeded to characterize the interaction between E6_M2_T2 and human IgG by MST (Figure 4D). We verified that using standard buffer conditions we could dissolve E6_M2_T2 and we obtained a dose response curve. Several parameters were estimated automatically from the equipment software. The EC_50_ was established as 157 nM and the Hills coefficient is 0.70, suggesting a non-cooperative binding. The estimated dissociation constant, K_D_ is 62 ± 14 nM. This value is half of the dissociation constant estimated for the E6 native sequence, indicating that the introduction of mutations and a tandem sequence increases the binding towards the target. The estimated dissociation constant is within the range of other affinity ligands, which shows the success of WW domains scaffold as affinity reagents (Kruljec and Bratkovič, 2017).

Finally, we characterised the secondary structure of the purified E6_M2 and E6_M2_T2 through ATR-FTIR (Supplementary Figure S9), and we observed the presence of 4 peaks - ∼1613 cm^−1^, ∼1626 cm^−1^, ∼1645 cm^−1^ and ∼1673 cm^−1^. The peak at ∼1613 and ∼1626 cm^−1^ can be assigned to β-sheets, the ∼1673 cm^−1^ can be assigned to β-turns, which are formed after each β-sheet and loop. The peak at ∼1645 cm^−1^ could be assigned to random coil, which can be associated with the N and C-terminal regions (Yang et al., 2015). These secondary structures were observed before in native and mutated WW domains (Davis and Dyer, 2014), thus indicating that the mutations in the E6 structure and production of a tandem sequence did not affect the secondary structure of the scaffold. The experimental results can be correlated with the predicted secondary structure from AlphaFold (Figure 4B; Supplementary Figures S10, S11), where the monomeric and tandem version have an average pLDDT >75% indicating a high confidence in the predicted structure showing secondary structures characteristic of WW domains and interestingly the tandem sequence is expected to organize as a V-shape structure.

## 4 Conclusion

The discovery of a novel affinity reagent towards human IgG using an artificial WW domain scaffold is reported. One lead ligand E6 was selected, due to its hydrophilic residues in loops and high signal in ELISA and similar amino acid composition in the loops as other human IgG affinity ligands reported in the literature. Production of E6 was optimized by supplementation of 200 mM L-arginine during expression up to 117 mg protein per L of culture. After purification, the dissociation constant was estimated as 133 ± 48 nM. We further explored the introduction of specific mutations in the framework of the WWp5_4 scaffold to improve the solubility and reduce chemical liabilities. This rational strategy resulted on the mutated scaffold WWp5_4_M2. The same strategy was applied to the E6 framework resulting in the sequence E6_M2. This was produced in the soluble form at 5-times high amounts than E6, while maintaining the binding activity against the target. Finally, we show that a tandem dimeric sequence (E6_M2_T2) aggregates less than the monomeric version and it can be consistently produced with 623 ± 56 mg of soluble protein per L of culture. After purification we could achieve a final purity of 90%. Furthermore, the MST experiments reveal a dissociation constant of 62 ± 14 nM, which is half of the value obtained for the E6 sequence. This improvement in scaffold developability, along with the sub-micromolar affinity for human IgG substantiates the approach of producing tandem sequences as an interesting strategy to improve affinity ligands, as reported before for Z domain derived from SpA. In the future, we consider that E6_M2_T2 can be further explored to interact with: fragments and full-length IgG from different species (e.g., mouse, goat); avian IgY, as we showed before for a synthetic ligand developed in house (Matos et al., 2021); or different immunoglobulin, e.g., IgA and IgM by cross-reactivity assays to determine the specificity. The relevance of cross-reactivity assays is highly dependent on the application (e.g., purification, *in vitro* diagnostic, therapeutics, etc), therefore should be considered as new research. Besides, ligand E6_M2_T2 could be conjugated with other molecules, e.g., fluorophores and proteins, as well as in solid-phase matrices through standard conjugation chemistry (namely, by maleimide-based chemistry), which will open various research avenues. Finally, this work further validates the use of the new WW domain scaffold for producing affinity reagents against different targets, thus expanding the range of possible applications in various biotechnological contexts.

## Data Availability

The original contributions presented in the study are included in the article/Supplementary Material, further inquiries can be directed to the corresponding author.
