# Supplementary material for: Discovery and optimization of a synthetic small protein domain targeting antibodies
Source: Front Bioeng Biotechnol. 2025 Nov 12;13:1678111. doi: 10.3389/fbioe.2025.1678111 (PMC12647017; doi:10.3389/fbioe.2025.1678111)
Supplement: Supplementary file 1 [file Supplementaryfile1.docx]

Supplementary Material

**Discovery and optimization of a synthetic small protein domain targeting antibodies**

Ana Margarida Gonçalves Carvalho Dias^1,2§^ , Manuel João Brandão Matos^1,2§^, Cátia Soares^1,2^, Carolina Natal^1,2^, Ana Sofia Pina^1,2^, Ana Cecília Afonso Roque^1,2^*

§1st co-authorship, authors contributed equally

^1^Associate Laboratory i4HB - Institute for Health and Bioeconomy, Chemistry Department, School of Science and Technology, NOVA University of Lisbon, Campus Caparica, 2829-516 Caparica, Portugal;

^2^UCIBIO – Applied Molecular Biosciences Unit, Department of Chemistry, School of Science and Technology, NOVA University Lisbon, 2829-516 Caparica, Portugal;

*Corresponding authors: [cecilia.roque@fct.unl.pt](mailto:cecilia.roque@fct.unl.pt)

Keywords (up to 5): phage-display; rational design; protein engineering; tandem domains; WW domains

1. **Supplementary Materials and Methods**

**1.1 Materials**

All used reagents were of the highest quality available and molecular biology grade. The SOB Broth, LB Broth, NZYStar competent cells, GreenSafe Premium, NZYDNA Loading Dye, NZYGelpure, NZYTech Miniprep, NZYDNA Ladder III, NZYDNA Ladder V, agarose, agar, glycine, tris base, ampicillin, kanamycin, tetracycline, sodium chloride, dNTPs NZYSet and *E. coli* BL21(DE3) competent cells were purchased to NZYTech. Precision Plus Protein^TM^ Dual Xtra prestained protein standard, 10% SDS solution, tricine, 30% Acrylamide/bis-acrylamide solution 19:1, 30% Acrylamide/bisacrylamide solution 29:1, 30% Acrylamide/bis-acrylamide solution 37.5:1, Fixative Enhancer Concentrate, Silver Complex Solution, Reduction moderator solution, Image development reagent, were purchased from Bio-Rad. 96-well 2mL deep round bottom blocks, Tetramethylethylenediamine (TEMED) and ammonium persulphate was purchased from Roth. The enzymes HindIII, NheI, Phusion HotStart II High-Fidelity DNA Polymerase, FastAP Thermo Alkaline Phosphatase and T4 Ligase were purchased from ThermoScientific. Standard 96-well microplates, Nunc Immuno Maxisorp ELISA plates, 1-StepTM TMB-Blotting Substrate, Spectra/Por 7 dialysis tubing 3kDa MWCO and anti-6x-HisTag-HRP monoclonal antibody, were purchased from ThermoFisher Scientific.

Human polyclonal IgG was purchased from Octapharma. Tris, fluoresceine isothiocyanate (FITC), sodium phosphate dibasic heptahydrate and sodium phosphate monobasic, coomassie brilliant blue R-250, ethylenediamine tetraacetic acid (EDTA), Tween20 detergent, glycerol, urea were purchased from Sigma. Amicon ultra centrifugal filters 30, 10 and 3 kDa and *E. coli* BLR cells were purchased from Merck Millipore. Forward and reverse primers were purchased from Eurofins Genomics and have the following sequences. Forward primer with NheI restriction site 5’ Aatagctagcatgtct atgggtctgccgccg 3’; Reverse primer with HindIII restriction site 5’ agacaagctttcaagaagacatacgcgggtcgg 3’; Reverse primer with SalI restriction site 5’ caatgtcgactcaagaagacatacgcgggtcgg 3’. The primer used for sequencing was pComb forward primer 3F 5’ ATGAAAAAGACAGCTATCGCGATTGCA 3’. HiTrap Phenyl FF Low Sub, HiTrap Q HP, HiTrap SP HP, HiLoad Superdex 75 pg and Sephadex G-25 PD-10 desalting column were purchased from GE Healthcare.

*E. coli* SS320, phagemid vector pComb3xSS, helper phage VCSM13, *E. coli* TOP10F’, *E. coli* ER2738, plasmid pET21c pAp006, *E. coli* Rosetta (DE3) competent cells, were available in-house and were used for the phage display and protein expression.

- 1. **Methods**

**Amplification Helper Phages VCSM13**

Each round of panning began with freshly prepared helper phages VCSM13. For that purpose, one aliquot of *E. coli* ER2738 was inoculated in 5mL of LB media with tetracycline (final concentration 10 µg/mL) at 37 ºC, 250 rpm until OD_600nm_ close to 1. After that, a mixture of 50 µL of cells with several dilutions of VCSM13 stock (ranging from 10^3^ to 10^12^) was incubated during 15 minutes at Room Temperature (RT). Each mixture was then transferred to a 15 mL Falcon containing 3 mL of Top-Agar (7 g/L of Agar, 25 g/L of LB) and dispersed to pre-warmed LB Agar plates (15 g/L of Agar, 25 g/L of LB) and then incubated overnight at 37ºC.

The following day another aliquot of *E. coli* ER2738 was inoculated in 10 mL of LB-Broth with tetracycline and was grown until OD_600nm_ reached 0.3. A phage colony from the overnight plates was picked and inoculated in the bacterial culture and was incubated during 2 hours at 37 ºC. After that time, those 10 mL were transferred to a 2 L Erlenmeyer flask with 500 mL of LB-Broth supplemented with 70 µg/mL kanamycin, 10 µg/mL tetracycline and was grown overnight at 37 ºC. The next day the culture was centrifuged at 2500 x g, 15 minutes at 4 ºC and the supernatant was kept and heated at 70 ºC for 20 minutes to kill the remaining bacteria. The solution was centrifuged again at 2000 x g, 20 minutes at 4 ºC and the supernatant was kept in aliquots of 40mL to be used in the phage display panning.

**Phage Precipitation and Pre-Panning**

For the first round of phage display panning the strain containing the WWp5_4 domain library (Dias et al., 2025) was amplified. For that purpose, one aliquot of *E. coli* SS320 containing the phagemid vector pComb3xSS (which contains the WW domain library) was inoculated in 500 mL of SOB medium supplemented with 10 mM MgCl_2_, tetracycline (final concentration 10 µg/mL) and ampicillin (final concentration 20 µg/mL) for 1 hour at 37 ºC at 220 rpm. After that, ampicillin was added up to a final concentration of 50 µg/mL and the culture was incubated when OD_600nm_ was 0.6-0.7. The culture was then infected with 1 mL of the helper phage stock VCSM13 and was incubated at 37 ºC for 2 hours. Finally, kanamycin was added to a final concentration of 70 µg/mL and was incubated overnight at 37 ºC.

The following day the culture was centrifuged at 2500 x g, 15 min at 4 ºC. The pellet was resuspended in 10% (v/v) glycerol and stored at -80 ºC. The pellet contained the phagemid DNA library transformed in the cells. The phage supernatant was harvested by precipitation with a 4% solution (w/v) of PEG-8000 / 3% solution (w/v) of NaCl during 45 minutes on ice. The solution was then centrifuged at 8000 x g, 20 minutes at 4 ºC. The pellet was resuspended in 1.5 mL of 1% (w/v) BSA in TBS and was centrifuged at 16500 x g, 5 minutes at 4 ºC. The phage supernatant was filtered at 0.22 µm and it was used for the first round of panning (Input titration sample).

**Small Scale Protein Expression and screening ELISA:** From the output of the 6^th^ round of panning, 94 isolated colonies were peaked into two sterile 96-well deep well 2mL round-bottom plates and grown with 200 µL of LB with ampicillin, 125 rpm, 37 ºC, overnight (master plates). The following day, to one of the master plates was added glycerol to achieve a final concentration of 15% and was stored at -80 ºC. Regarding the other master plate, 12 µL of this culture was added to another sterile 96-well deep well 2 mL round-bottom plate with 1.2 mL of LB with ampicillin and was grown at 37 ºC, 125 rpm until OD_600nm_ achieved 0.5-0.6. After this, protein expression was induced with 1 mM IPTG and continued incubation with the previous conditions. The following day, the cultures were sonicated for 30 minutes in ice-water bath and centrifuged for 10 minutes at 2500 x g. The supernatant was collected and transferred to a new microplate to be used in the screening for the ELISA (crude extract). For the ELISA, three microplates were tested: A) Sample binding plate; B) Negative control plate; C) Expression plate. On the sample binding plate, 1 µg/well of human polyclonal IgG was immobilized on the plate at 4 ºC, overnight. On the following day, the target was removed, and the wells were washed once with TBS, then the plate was blocked with 300 µL 3% (w/v) BSA in TBS for 1 hour at 37 ºC. After this, the wells were washed once with TBS, and were then incubated with 50 µL of crude extract (containing WW domain) for 1 hour at 37 ºC. The plate was then washed 5 times with TBS-T, and then incubated with 100 µL of the Anti-HA tag-HRP conjugated antibody (diluted 1:1000) for 1 hour at 37 ºC. The plate was then washed once with TBS-T and 4 times with TBS, and then incubated with the substrate ABTS for 1 hour at the dark at 37 ºC. The results were then analysed in a microplate reader (TECAN) and the Abs_405nm_ was then measured.

For the Negative control plate the protocol was the same as for the Binding microplate, except for the target immobilization. Instead of having IgG, only coating buffer was added. This plate allows to detect clones that unspecific bind to BSA present in the Blocking Buffer. For the Expression plate the protocol started at the step of incubation with the crude extract, and from there the steps were the same as the Binding microplate. This plate allows to detect clone’s basal expression level.

After analysing the ELISA results the best clones were sent for sequencing. The plasmid DNA was purified following manufacturer’s instruction using NZYMiniprep kit. The pure plasmids were sequenced in Eurofins Genomics using the pComb forward primer 3F (5’ ATGAAAAAGACAGCTATCGCGATTGCA 3’). The resulting sequences were aligned using ClustalW2 and analysed using Unipro UGENE.

**E6 gene cloning:** The insert corresponding to the chosen E6 WW clone was amplified using Polymerase Chain reaction (PCR) with the designed forward and reverse primer (SalI). The reaction was set using the Phusion Hot Start II High Fidelity DNA Polymerase according to manufacturer’s indication. The PCR mixture contained 4 µL of 5x Phusion GC Buffer, 0.4 µL of each dNTP (10 mM stock), 0.5 µL of each primer (50 µM stock), 1ng purified plasmid, 0.6 µL DMSO, 0.2 µL Phusion (1:2 dilution of stock) and sterile MilliQ water to fulfil 20 µL of total volume. The conditions for the reaction were as follows: 98 ºC for 30 s; 25 cycles of 98 ºC for 10 s, 72 ºC for 1 min 11 s; 72 ºC for 10 minutes. The vector’s DNA, pET21C pAP006 was double digested using NheI and SalI (3 hours at 37 ºC, 10 minutes at 65 ºC followed by 5 minutes at 80 ºC) and it was further dephosphorylated using 5 µL FastAP (30 minutes incubation at 37 ºC and 20 minutes at 80 ºC for inactivation). The expected DNA fragments were analysed on a 2% (w/v) and 0.8% (w/v) agarose gel (100V, 1 hour) and extracted following NZYGelpure manufacturer’s protocol.

For the transformation, 50 µL of competent cells (NZYStar) and 5 µL of ligated product were mixed and incubated on ice for 30 minutes. After this incubation period, the mixture was subjected to a heat-shock treatment for 40 seconds at 42 ºC and then incubated in ice for 2 minutes. The reaction volume was fulfilled to 1mL with LB medium and incubated at 37 ºC for 1 hour at 210 rpm. Afterwards, 50 µL of transformed cells were spread over LB-agar ampicillin plates and incubated at 37 ºC overnight. The transformation protocol included a negative control (no plasmid + 50 µL competent cells) and a positive control (1 µL of competent cells control plasmid + 50 µL of competent cells). Individual clones were then picked for restriction analysis by colony PCR. The desired individual clones were picked with a sterile tip and dipped in a PCR tube containing 10 µL of sterile MilliQ water. This mixture was then incubated for 10 minutes at 98 ºC. Afterwards, the reagents added and protocol was the same as for standard PCR. The DNA was then loaded onto a 2 % (w/v) agarose gel to analyse presence of the insert DNA (100V, 1 hour).

**Purification of E6 sequence**

Ion-exchange chromatography (IEX) was performed using HiTrap SP HP (17115101, Cytiva). The column was previously equilibrated with 10 column volumes (CV) with binding buffer. The supernatant was diluted in urea and loaded onto the column and afterwards it was washed with 10 CV of binding buffer, followed by gradient elution in 10 CV of 0-50% of elution buffer followed by 5 CV of 50-100% of elution buffer. The tested binding buffers were: i) 50 mM MES pH 6; and ii) 50 mM MES with 4 M Urea pH 6, while for elution were used: i) 50 mM MES with 1 M NaCl pH 6; and ii) 50 mM MES with 4M Urea and 1M NaCl pH 6.

Size-exclusion chromatography (SEC) was performed using HiLoad Superdex 75pg (28989333, Cytiva). The column was previously equilibrated with 2 CV with binding buffer (PBS) and the sample was loaded onto the column through the injection valve on a capillary loop. Elution fractions of 1mL of all techniques were collected and analysed in terms of the protein quantification and samples purity and the fractions containing most of the WW protein were pooled. All the chromatographic techniques were performed on ÄKTA Pure automated system.

For buffer exchange, dialysis with Spectra/Por 7 dialysis tubing 3kDa MWCO were used. The proteins fractions were contained in the dialysis tubing with buffer (in >100x ratio) and were gently stirred at 4ºC. The buffer was changed every 3 hours for 2 days to achieve total exchange of ionic species. For protein concentration Amicon Ultra-15 3kDa MWCO were used with cycles of 4900 x g for 30 minutes at 4ºC.

**Protein quantification**

The quantification of protein present in the samples was performed using bicinchoninic acid (BCA) assay reagent provided by Thermo Scientific Pierce, using a protein standard of bovine serum albumin (BSA). The samples were analysed at 562 nm in microplate reader Tecan Infinite F200 to create a linear regression curve.

**Protein gel electrophoresis**

The samples were prepared for electrophoresis by mixing in equal amounts with sample buffer (100 mM Tris-HCl pH 6.8, 24% (v/v) glycerol, 1% (v/v) SDS, 4% (v/v) β-mercaptoethanol, 0.02% (w/v) bromophenol blue). The samples were applied onto Tris-Tricine gels and ran at 100V for 3 hours with electrophoresis buffer (25mM Tris, 25mM Tricine, 0.05% (w/v) SDS). The gel was transferred onto a staining solution (1g Coomassie Blue R-250, 15mL acetic acid, 90mL methanol and 95mL distilled water) for 30 minutes. The gel was then transferred onto a destaining solution (same composition as staining solution without Coomassie Blue R-250). When necessary, Sodium dodecyl sulfate–polyacrylamide gel electrophoresis (SDS–PAGE) was also performed to evaluate the samples purity in a 12.5% acrylamide gel and ran at 120V. Images were acquired with Gel DocTM XR + System with Image Lab^TM^ software from Bio-Rad and analyzed with ImageJ software.

**Supplementary Figures and Tables**

**Table S1** - Conditions used for the biopanning scheme against human IgG.

| **Parameter** | **1^st^ Round** | **2^nd^ Round** | **3^rd^ Round** | **4^th^ Round** | **5^th^ Round** | **6^th^ Round** |
| --- | --- | --- | --- | --- | --- | --- |
| IgG concentration (nM) | 222 | 111 | 22.2 | 11.1 | 11.1 | 11.1 |
| Number of washes | 5 | 10 | 15 | 15 | 15 | 15 |
| Incubation time and temperature | 2 hours, 22 °C | | | | | |

**Table S2** - Results obtained for the input and output titration of the biopanning.

| **Panning Round** | **Input (pfu/mL)** | **Output (pfu/mL)** |
| --- | --- | --- |
| 1st | 1.3x10^14^ | 8.9x10^7^ |
| 2nd | 4.1x10^13^ | 1.6x10^5^ |
| 3rd | 1.4x10^15^ | 1.0x10^5^ |
| 4th | 6.5x10^14^ | 2.0x10^6^ |
| 5th | 1.0x10^14^ | 3.4x10^7^ |
| 6th | 1.5x10^15^ | 3.5x10^8^ |

**Figure S1** – Analysis of the relative amino acid distribution (in %) in Loops I and Loop II per group

**Figure S2** - Evaluation of the expression of the selected clones at small scale. Tris-Tricine gels. BI- Before induction; AI- After induction; MM- Molecular weight marker (Precision Plus Protein™ Dual Xtra Prestained, Biorad). Stained with Silver staining.

**Table S3** - Biological production tests for optimization of protein production.

| **Test** |  | **Bacterial culture conditions** |
| --- | --- | --- |
| No supplementation | Test A | No supplementation after induction |
| Glutamate supplementation | Test B | Supplementation 50mM L-glutamic acid after induction |
| Arginine supplementation | Test C | Supplementation 50mM L-arginine after induction |
|  | Test D | Supplementation 100mM L-arginine after induction |
|  | Test E | Supplementation 200mM L-arginine after induction |
| Amino acids Mixture | Test F | Supplementation 50mM L-arginine and L-glutamic acid after induction |
|  | Test G | Supplementation 100mM L-arginine and L-glutamic acid after induction |
|  | Test H | Supplementation 200mM L-arginine and L-glutamic acid after induction |
| Growth until OD 0.6-0.8, induction with 1mM IPTG incubation at 30 ºC for 20h | | |

**Figure S3** - Tris-Tricine gels of the time-course expression of E6 in different amino acid supplementation conditions in the culture media. Test A - No supplementation after induction; Test B - Supplementation 50mM L-glutamic acid after induction; Test C to test E – Supplementation with increasing concentrations of L-arginine after induction (50mM; 100mM; 200mM); Test F to test H – Supplementation with increasing concentrations of a mixture of L-arginine/L-glutamic acid after induction (50mM; 100mM; 200mM). BI- Before induction; MM- Molecular weight marker (Precision Plus Protein™ Dual Xtra Prestained, Biorad). Stained with silver staining.


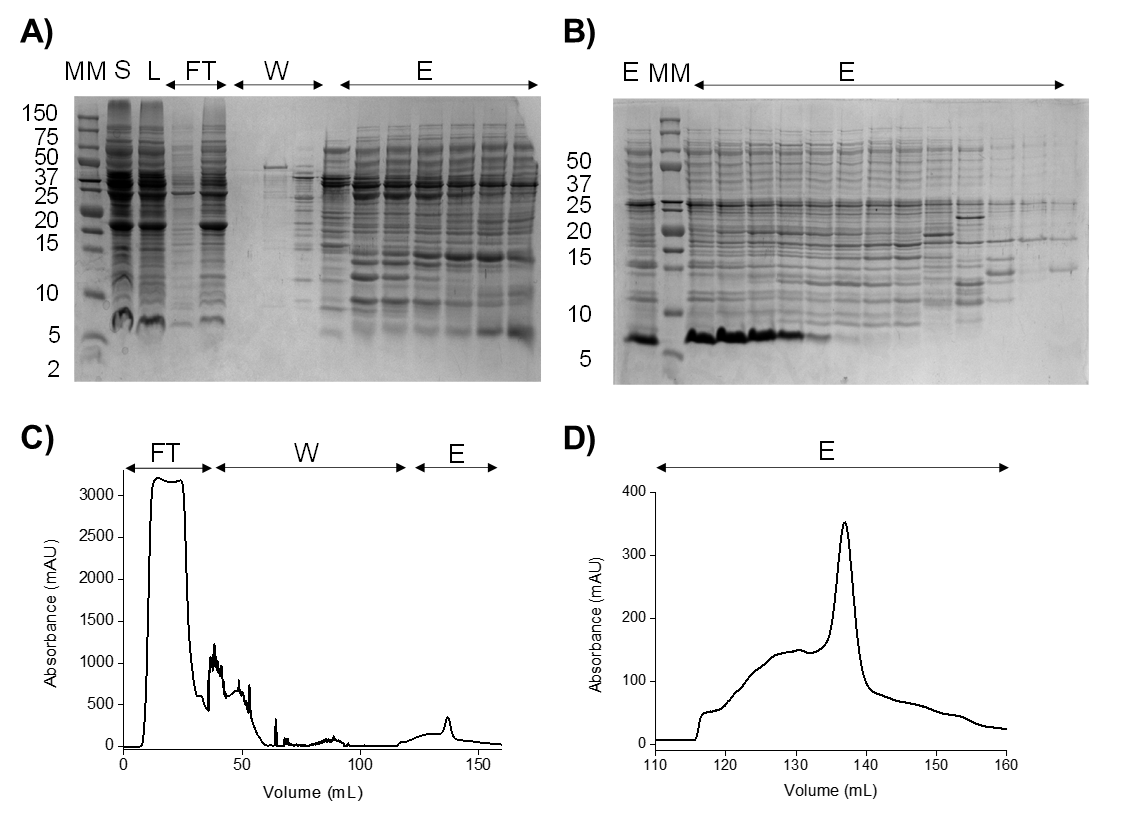


**Figure S4** - Tris-Tricine gel (A and B) and chromatogram of E6 purification on a cation-exchange chromatography with supplementation of 4M urea (C and D). A and B show the electrophoresis gel of fractions collected during the purification run. C – Chromatogram of the purification and D - Chromatogram zoom in in the Elution fractions. The major Elution peak represent the E6 fraction. MM- Precision Plus Protein™ Dual Xtra Prestained, Biorad, L- Load of the protein mixture to the column, FT- Flowthrough, S- Cell culture soluble supernatant, W- Wash of the column with binding buffer, E- Elution with 50mM MES 1M NaCl and 4 M urea pH 6. Stained with silver staining.

**Figure S5** - Lead ligand purification by Size Exclusion Chromatography. Chromatogram (A) and tris-Tricine gel of E6 purification on SEC (B,C). MM- Precision Plus Protein™ Dual Xtra Prestained, Biorad; P- Pool IEX chromatography; C- Concentrated pool IEX with Amicon; Perm - Permeate of concentration with Amicon; L - Loading in SEC; V0- Fraction of the void volume of the volume; Ve- Fractions of the volume of elution of the column. Stained with silver staining.

**
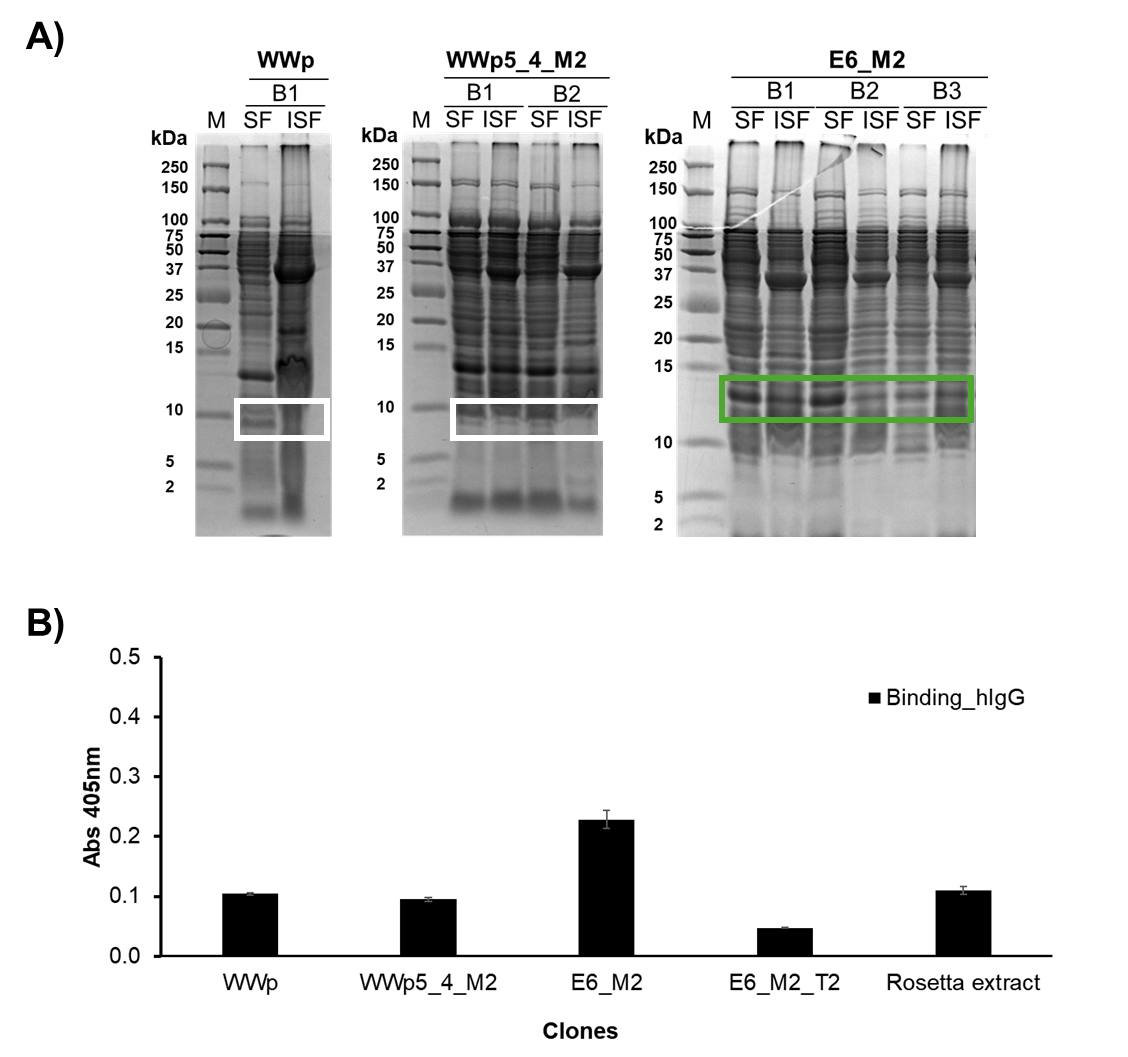
**

**Figure S6** – Production and signal characterization of mutated versions. A – Tris-Tricine gels after cell lysis of the WW prototype sequence (WWp, band at ~8KDa), mutated WWp5_4 scaffold (WWp5_4_M2, band at ~8 kDa) and mutated E6 (E6_M2, band at ~ 12kDa). Samples were normalized 90µg total protein/lane. M - Precision Plus Protein™ Dual Xtra Prestained, Biorad; SF – soluble fraction; ISF – insoluble fraction. B1, B2, and B3 indicate production batchs in 1L culture, except for WWp B1 – 100 mL, WWp_5_4 B2 – 900 mL. Stained with Simplyblue. B – ELISA of the different clones against human IgG (hIgG). Extract from *E.coli* Rosetta (DE3) was used as negative control for binding against the target. The detection of the clones was made with anti-His conjugated antibody with HRP and detected with ABTS as substrate.

**
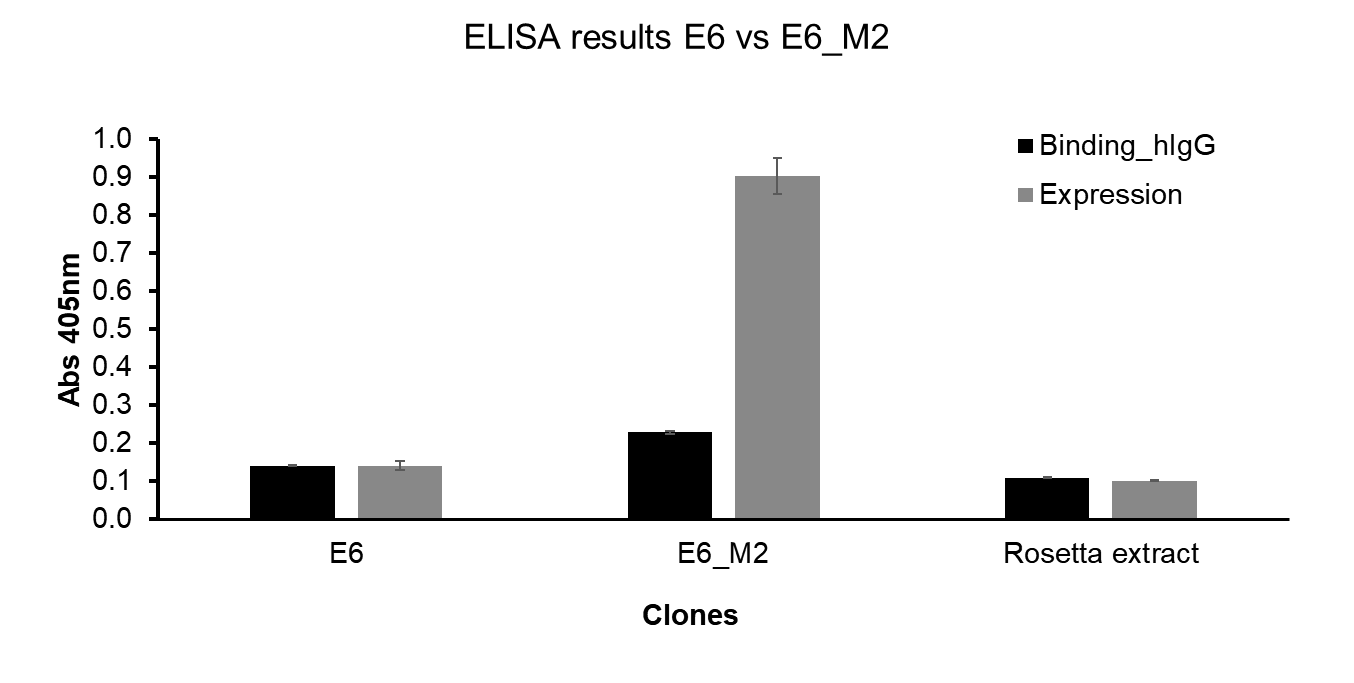
**

**Figure S7** – Comparison between ELISA signal of E6 versus E6_M2. ELISA of the different clones against human IgG (hIgG). E6 was produced in pComb3x vector (Hemaglutin tag) and the E6_M2 was produced in pET15b vector (His tag). Extract from *E.coli* Rosetta (DE3) was used as negative control for binding against the target. The expression levels were detected by direct coating of the microplate well with the soluble fraction of the extract. The detection of the clones was made with E6 with anti-HA conjugated antibody HRP and E6_M2 and Rosetta extract with anti-His conjugated antibody with HRP. All were detected with ABTS as substrate.


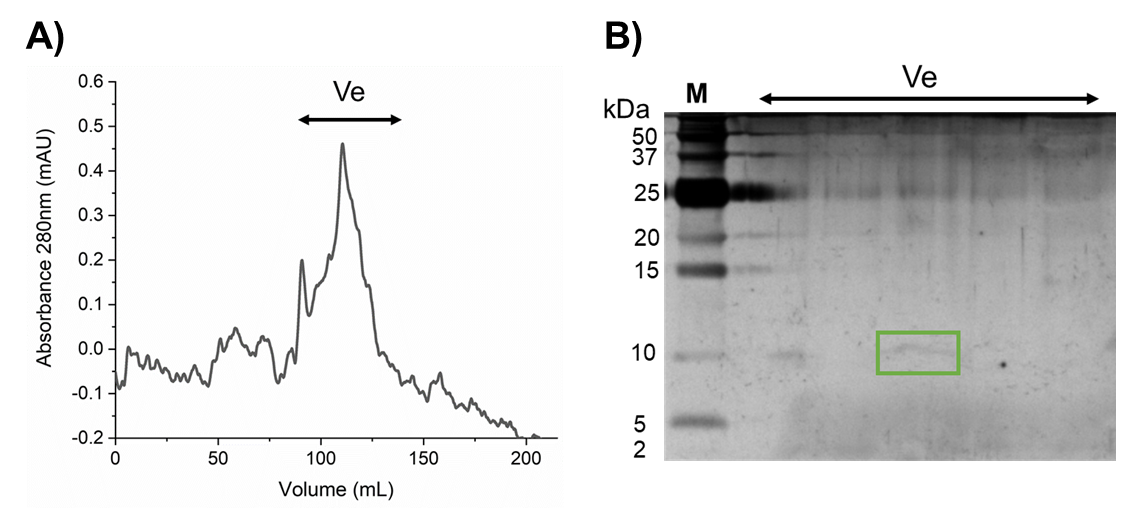


**Figure S8** – Purification of E6_M2_T2 by Size Exclusion Chromatography (SEC). A – Chromatogram. B – Tris-Tricine gel of collected fractions after SEC Samples were normalized 1µg total protein/lane. Ve- Fractions of the volume of elution of the column. M - Precision Plus Protein™ Dual Xtra Prestained, Biorad. Stained with Silver Staining.

**
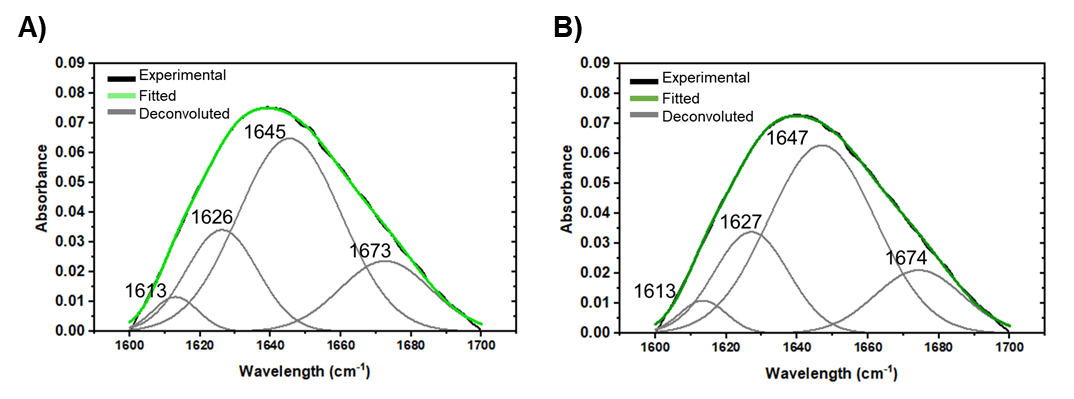
**

**Figure S9 -** Attenuated total reflectance – Fourier transform infrared spectroscopy (ATR-FTIR) analysis of: A) E6_M2 and B) E6_M2_T2. Representation of the Amide I band using peak deconvolution tool from Origin2024 software.

**
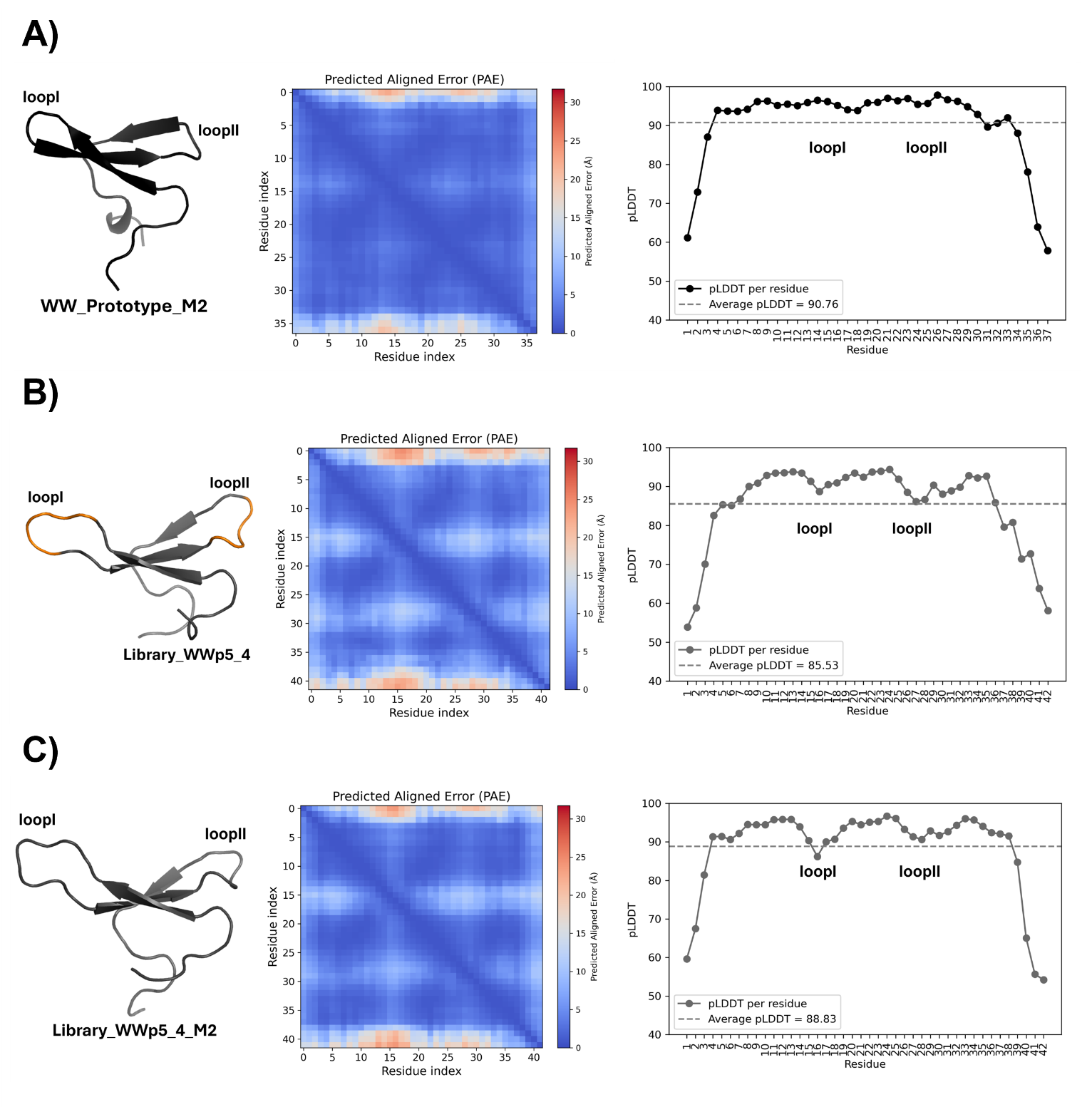
**

**Figure S10 –** Predicted structures of WW library generated by AlphaFold3. The models and data reports are displayed for each structure (graphs: Predicted aligned error (PAE) and the pLDDT per residue). A) The WW prototype mutated structure has an average pLDDT of 90.76. B) The WWp5_4 model structure as an average pLDDT of 85.53, where in orange is identified the randomized loops and in grey is represented the framework maintained constant. C) The WWp5_4_M2 structure has an average pLDDT of 88.83. The pLDDT improves as the mutations are incorporated in the library sequence, indicating a reliable predicted structure. Images were generated in Pymol software.

**
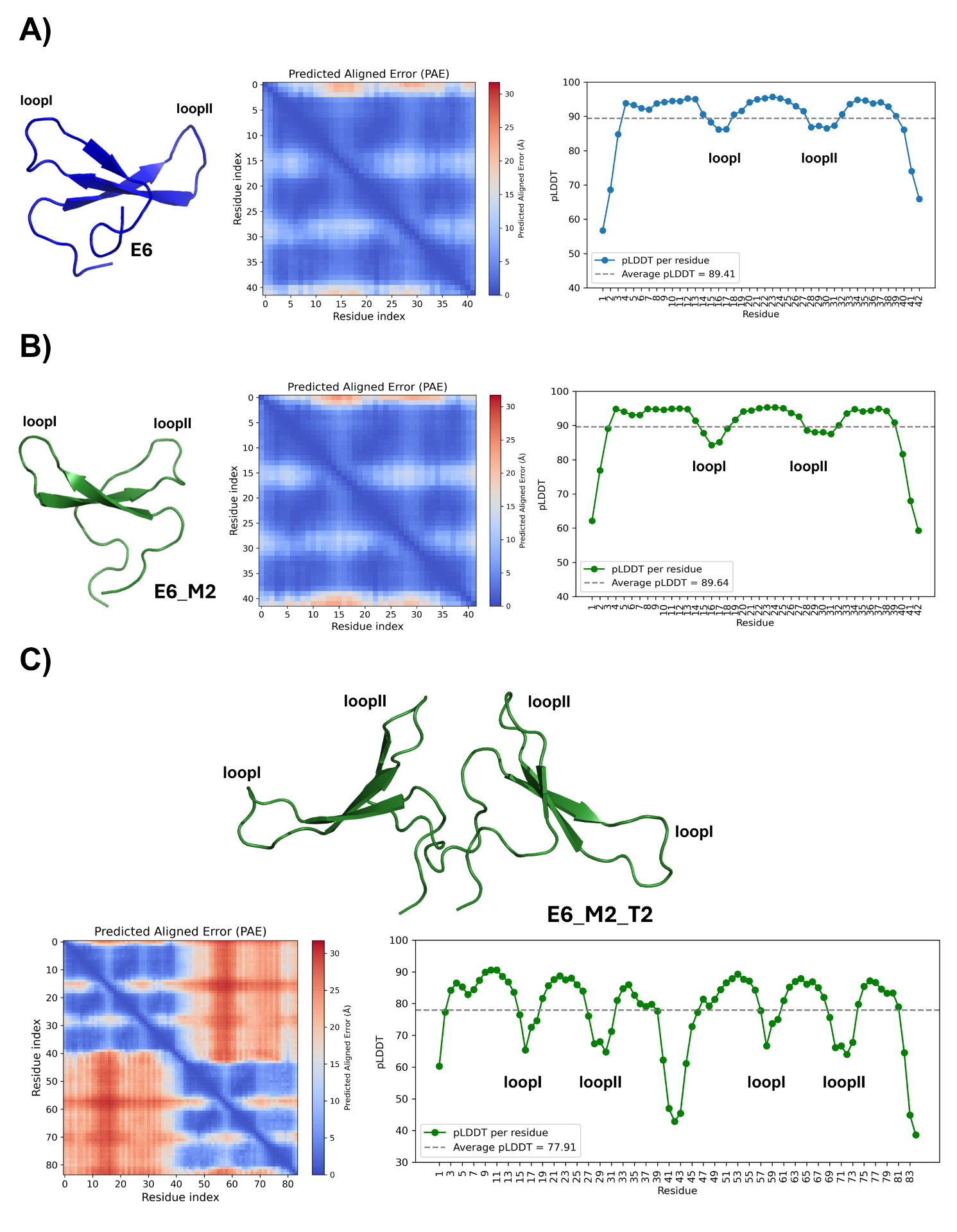
**

**Figure S11 –** Predicted structures of E6 lead ligand and mutates generated by AlphaFold3. The models and data reports are displayed for each structure (graphs: Predicted aligned error (PAE) and the pLDDT per residue). A) The E6 structure has an average pLDDT of 89.41. B) The E6_M2 model structure as an average pLDDT of 89.64. The pLDDT improves as the mutations are incorporated in the E6 sequence, indicating a reliable predicted structure. C) The tandem sequence – E6_M2_T2 structure has an average pLDDT of 77.91, this is due to the large loop sequences that might contribute to have partially unfolded regions. Images were generated in Pymol software.

**References:**

Dias, A. M. G. C., Teixeira, G. D. G., Barbosa, A. J. M., Goncalves, J., Iranzo, O., and Roque, A. C. A. (2025). Design and evolution of a synthetic small protein scaffold based on the WW domain. *Protein Science* 34, e70164. doi: https://doi.org/10.1002/pro.70164
